# Supplementary material for: Efficacy of Cognitive Behavior Therapy in Reducing Depression among Patients with Coronary Heart Disease: An Updated Systematic Review and Meta-Analysis of RCTs
Source: Healthcare (Basel). 2023 Mar 24;11(7):943. doi: 10.3390/healthcare11070943 (PMC10094182; doi:10.3390/healthcare11070943)

SUPPLEMENTARY FILES

Table S1. Characteristics of Selected Studies

	First Author; Year; Country; Study Design	Population	Sample	Male (%)	Age (y)	Instruments	Intervention	Comparison	Outcomes (Depression)	Appraisal Results
1	Turner 2013<br>New Zealand<br>A single-blind RCTs	Participants aged ≥ 18 years, diagnosed with CHD for at least two months (Coronary Syndrome (ACS); undergoing PCI; undergoing Coronary Artery Bypass Graft (CABG) and/or a definite diagnosis of a heart condition, and experiencing depression as measured by BDI II.	57 patients.<br><br>Intervention n=25<br><br>Control n=32	Intervention: 76%<br><br>Control: 72%	Intervention: 61±11<br><br>Control: 62±9	BDI-II	Intervention delivered by psychologist, 6 session (1,5h/session), a face to face group therapy other therapy outside study were allowed.	Intervention: Brief intervention and CBT<br>Control: Brief intervention	Meanscore:<br>Intervention group:<br>Baseline: 25.7(7.33)<br>2 mo: 20.5(8.4)<br>6 mo: 19.8(10.3)<br>12 mo: 17.4(10.3)<br><br>Brief session (CG):<br>Baseline: 27.2(8.32)<br>2 mo: 19.6(9.3)<br>6 mo: 17.8(9.5)<br>12 mo: 16.6(10.5)<br><br>There is no significant difference in all time points between the control and intervention groups (baseline, 2, 6, and 12 months) p>.05	92%
2	Davidson 2013<br>USA<br>Multi-center randomized controlled trial	Participants were patients with elevated depressive symptoms; 2 to 6 months after an ACS.	150 patients.<br><br>Intervention n=73<br><br>Control n=77	Intervention 59%<br><br>Control 57%	Intervention 59.2±9.7<br><br>Control 60±11.1	BDI-II	A face to face individual and combine therapy, Intervention delivered by therapist, psychiatry, psychology supervisor and registered nurse.	Intervention: PST, medication (sertraline hydrochloride, citalopram hydrobromide)<br>Control: Usual care	Intervention group<br>6 mo: 24(47.1)<br>Δ -10.1 (95%, CI -12 – 8.1)<br><br>Usual Group (U.C.)<br>6 mo: 16(27.6)<br>Δ -6.6 (95%, CI -8.5 – (-4.8)<br><br>The active treatment group had a greater reduction in BDI-scores (differential change between groups -3.5 (95% CI -6.1 to -0.7; p=.01).	100%
3	Doering 2013<br>RCTs<br>USA	Patients with depressive symptoms. Age > 30 y, one month	81 patients post cardiac surgery	Intervention n=80%	Intervention n=63.4±8.4	BDI-II	intervention delivered by advanced practice nurses, 8 session (50-	Intervention: CBT<br>Control:	The intervention was effective in improving depression particularly for those who received early CBT (β=0.79[95% CI 0.10 – 1.47]).	85%

	First Author; Year; Country; Study Design	Population	Sample	Male (%)	Age (y)	Instru ments	Intervention	Comparison	Outcomes (Depression)	Appraisal Results
		after cardiac surgery procedures.	(CABG, AVR, MVR).<br><br>Intervention n=45.<br><br>Control: n=36.	Control n=55,6%	Control n=63.9±11.4		60 min /session) , A face to face individual therapy	Usual care. After completion of U.C. period, a control group were offered later CBT+UC (waitlist)	Intervention group:<br>8 weeks (2 mo): Δ -6.91(9.9)<br><br>U.C.:<br>8 weeks (2 mo): Δ 2.03(9.8)<br><br>Late CBT<br>8 weeks (2 mo): Δ -5.40(7.2)	
4	O'Neil 2014<br>Australia<br>A two-arm multicenter RCts	Patients ACS with depression who admitted to one of six hospitals.	121 patients<br><br>Intervention n=61<br><br>Control n=60	Intervention n=73.8%<br><br>Control n=76.7%	Intervention=61.0±10.2<br><br>Control= 58.9±10.7	PHQ9 and CDS	intervention delivered by psychologist, 10 session, telephone based individual therapy	Intervention: CBT<br>Control: Usual care	Intervention group:<br>PHQ9: Baseline: 9(5.4)<br>6 mo: 6.1(5.5)<br>Δ -3.00(4.30)<br>U.C.:<br>PHQ9: Baseline: 9.4(5.2)<br>6 mo: 8.11(5.8)<br>Δ -1.27(5.24)	92%
5	O'Neil 2015<br>Austalia<br>A two-arm multicenter RCts	Patients ACS with depression who admitted to one of six hospitals.	121 patients<br><br>Intervention n=61<br><br>Control n=60	Intervention n=73.8%<br><br>Control n=76.7%	Intervention=61.0±10.2<br><br>Control= 58.9±10.7	PHQ9 and CDS	intervention delivered by psychologist, 10 session, a telephone based individual therapy	Intervention: CBT<br>Control: Usual care	MDD patients<br>Intervention group (n=28)<br>PHQ9: 12 mo: 6.5(4.9-8.0)<br><br>UC (n=28):<br>PHQ9: 12 mo: 9.3(7.7-10.9)<br><br>No MDD patients<br>Intervention group<br>PHQ9: 12 mo: 6.2(4.8-7.5)<br><br>UC:<br>PHQ9: 12 mo: 5.2(3.9-6.6)<br><br>Overall, after 12 months<br>PHQ9: Intervention group had mean score 6.5; 95% CI: 4.9–8.0 versus Control group: 9.3; 95% CI: 7.7–10.9, p=0.012.	92%

	First Author; Year; Country; Study Design	Population	Sample	Male (%)	Age (y)	Instruments	Intervention	Comparison	Outcomes (Depression)	Appraisal Results
6	Fernandes 2017 Portugal RCTs	Patients with ACS in a central hospital.	121 patients.<br><br>Intervention n=65<br><br>Control n=56	Intervention n=68.8%<br><br>Control n=71.4%	Intervention =61.77±12.1<br><br>Control =66.11±12.6	HADS	intervention delivered by psychologist and cardiologist , 3 session (1h15min/session, a face to face group and combination therapy, added health education for CHD.	Intervention: CBT<br>Control: Usual care	Intervention group<br>Baseline: 12.8(4.05)<br>1 mo: 5.16(0.35)<br><br>U.C.:<br>Baseline: 9.61(4.49)<br>1 mo: 12.94(0.38)	72%
7	Norlund 2017 Sweden RCTs	Patients discharge after an Miocardial Infarction (MI), CABG and PCI.	362 patients.<br><br>Intervention n=192<br><br>Control n=170	Interventionn= 77.6%<br><br>Control n=75.3%	Intervention=62.0±7.94<br><br>Control=61.0±8.28	The Depressive Mood Scale and the Every day Life Stress Scale (ELSS)	intervention delivered by psychologist and nurse , 20 session (2 h/session), a face to face group therapy.	Intervention: CBT<br>Control: Usual care	Depression improved over time but there were no significant differences between groups.<br>No information regarding post-test Mean(S.D.) between I.G. and U.C.	100%
8	Norlund 2018 Sweden Multicenter RCTs	Patients with recent MI and were having depression or anxiety simptoms.	239 patients<br><br>Intervention n=117<br><br>Control n=122	Intervention n=62.4%<br><br>Control n=70.5%	Intervention=58.4±9<br><br>Control =60.8±7.8	HADS	Intervention delivered by psychologist, an internet and telephone-based therapy.	Intervention: iCBT<br>Control: Usual care	Intervention group<br>Baseline: 9.9(2.2)<br>14 weeks/±3 mo: 6.6(3.3)<br><br>UC:<br>Baseline: 10.3(2.5)<br>14 weeks/±3 mo: 8.0(3.8)<br><br>There was a reduction in HADS scores over time but no difference between the groups at follow-up (beta=−0.47, 95% CI −1.95 to 1.00, P=.53).	92%

	First Author; Year; Country; Study Design	Population	Sample	Male (%)	Age (y)	Instruments	Intervention	Comparison	Outcomes (Depression)	Appraisal Results
9	Rafanelli 2020<br>Italia<br>Two centers RCTs	Patients who were hospitalized for a first episode of AMI or unstable angina with depressed and/or demoralized conditions.	100 patients<br><br>Intervention n=50<br><br>Control n=50	Intervention n=62%<br><br>Control n= 76%	Intervention=57.64±9.99<br><br>Control n=60.02±10.94	The Structured Clinical Interview for Depression.	Intervention delivered by psychologist, 12 session (45m/session), a face to face individual and combination therapy, added Well being therapy intervention	Intervention: CBT+WBT<br>Control: Clinical management	Intervention group (The sequential combination of CBT/WBT)<br>Baseline: 7.92(4.77)<br>Post-test: 5.19(4.96)<br>3 mo: 6.38(5.03)<br>6 mo: 7.06(5.22)<br>12 mo: 6.91(5.08)<br>30 mo: 5.99(4.64)<br><br>UC(Clinical Management)<br>Baseline: 6.90(4.87)<br>Post-test: 5.94(4.22)<br>3 mo: 5.83(4.75)<br>6 mo: 6.80(5.45)<br>12 mo: 6.22(5.09)<br>30 mo: 5.83(4.18)	100%
10	Humphries 2021<br>Sweden<br>Multicenter RCTs	Patients with recent AMI reporting mild-to-moderate symptoms of anxiety or depression.	239 patients<br><br>Intervention n=117<br><br>Control n=122	Intervention n=62.4%<br><br>Control n=70.5%	Intervention 58.4±9<br><br>Control 60.8±7.8	HADS	Intervention delivered by therapist for 14 weeks, an internet and telephone-based therapy added.	Intervention: iCBT<br>Control: Usual care	Intervention group:<br>Baseline: 9.9(2.2)<br>12 mo: 6.2(3.8)<br>Δ -3.7<br><br>UC:<br>Baseline: 10.3(2.5)<br>12 mo: 7.8(3.5)<br>Δ -2.5	100%
11	Freedland 2009<br>USA<br>RCTs	Patients aged 21 years or older who had undergone CABG surgery within the past year.	Post CABG Patients<br>Intervention n=41<br><br>Control n=40	Intervention n=44%<br><br>Control n=57%	N/A	BDI II	Intervention delivered bt psychologist and social worker, 2 sesseion (50-60m/session), A face to face but sometimes use phone call therapy. Antidepressant medication was allowed as long as the patient had been	Intervention: CBT<br>Control: Usual care	Intervention group<br>Baseline=22.3(1.3)<br>3 mo= 5.4(1.3)<br>6 mo=7.8(1.3)<br>9 mo= 6.7(1.3)<br><br>UC<br>Baseline=20.8(1.4)<br>3 mo=13.8(1.4)<br>6 mo=10.7(1.4)<br>9 mo=12.9(1.4)<br><br>P between groups .02	92%

	First Author; Year; Country; Study Design	Population	Sample	Male (%)	Age (y)	Instruments	Intervention	Comparison	Outcomes (Depression)	Appraisal Results
							taking a therapeutic dose for at least 6 weeks			
12	Berkman 2003 USA Multicenter RCTs	All patients with an acute MI admitted to the participating hospitals (73 hospitals)	CHD depressed Patients, Intervention n=1238 Control n=1243	Intervention n=57% Control n=56%	Intervention 61±12.6 Control= 61±12.5	BDI II	Intervention delivered by therapist, The maximum duration of intervention was 6 months. Group therapy could extend an additional 12 weeks and adjunctive pharmacotherapy for up to 12 months, at which time the patient was reevaluated by the ENRICHD psychiatris, intervention group patient was initiated antidepressan (sertraline hydrochloride) 50mg/d and maximum 200mg/d as necessary, a mixed theraphy.	Intervention: CBT+ antidepressant Control: Usual care	Intervention group: Baseline: 17.7(8.1) 6 mo: 9.1(8.6) Δ -8.6 (9.2) U.C.: Baseline: 18.0(7.6) 6 mo: 12.2(9.1) Δ -5.8 (8.9) CI −2.7 (−3.7 to −1.7) (P<.001)	85%
13	Dao 2011 USA RCTs	Patients pre-post operative (CABG) with symptoms of depression or anxiety	Patients Pre-CABG. Intervention n=48 Control n =49	Intervention n=77.1% Control n=79.6%	Intervention= 62.8 ±11.8 Control= 64.2 ±11.9	BDI II	Intervention delivered by therapist, 2 sessions before surgery and 2 sessions after surgery (1h/session), A face to face individual therapy. Antidepressant	Intervention: CBTwith MADES (Managing Anxiety and Depression using Education and Skills) Control: Usual care	Intervention group: Baseline: 23(6.6) Post-test (1): 15.9(5.1) Post-test (2) 1 mo: 19.2(6.7) U.C.: Baseline: 22.4(6.2) Post-test (1): 23.4(11.6) Post-test (2) 1 mo: 22.5(10.7)	100%

	First Author; Year; Country; Study Design	Population	Sample	Male (%)	Age (y)	Instruments	Intervention	Comparison	Outcomes (Depression)	Appraisal Results
							medication was allowed as long as the patient had been taking a therapeutic dose for at least 6 weeks			
14	Nejati 2022<br>Iran<br>RCTs	Patients with CHD who were accepted to the private clinics of cardiologists of Mashhad (Iran) for coronary angiography.	CHD Patients. CBGI-CHD n=17<br><br>Cognitive Therapy in Groups (CTG) (CTG) of Type-D Personality n=17<br><br>Control Group (C.G.) n=17	N/A	CBGI-CHD= 59±10.48<br><br>CTG= 57±9.17<br><br>CG=58±9.77	BDI-II	Intervention delivered by psychologist and cardiologist, CBGI-CHD=12 session (2,5h/session) CTG 12=session (2h/session), A face to face group therapy	Intervention:<br>1. CBGI-CHD<br>2. CTG<br>Control:<br>Usual care	Intervention Group:<br>CBGI-CHD<br>Pre-test=21.46(5.74)<br>Post-test=18.88(5.26<br><br>CTG<br>Pre-test=22.81(6.14)<br>Post-test= 18.54(5.46)<br><br>UC:<br>CG<br>Pre-test= 21.16(8.50)<br>Post-test= 20.17(5.87)	75%
15	Zetta 2011<br>Scotland	Patients with anginal symptoms (cardiac disease, CHD)	Intervention n=103<br><br>control n=102	Male Intervention: n=72%<br><br>control n=65%	Intervention: 64.8 (10.04)<br><br>Contol: 65.94 (9.96)	HADS	Intervention delivered by nurse, incorporated to existing rehabilitation services. During a 45 min in hospital consultation the A.P. nurse completed an assessment and initiated the A.P. intervention, which was then facilitated over the next 12	Intervention:<br>CBT<br>Control:<br>Usual care	Intervention<br>Baseline: 2.07 (0.84)<br>6 mo: 2.00 (0.93)<br><br>Control<br>Baseline: 2.07 (0.79)<br>6 mo: 2.15 (0.86)	75%

	First Author; Year; Country; Study Design	Population	Sample	Male (%)	Age (y)	Instru ments	Intervention	Comparison	Outcomes (Depression)	Appraisal Results
							weeks, An individual telephone based therapy			
16	Barth 2005 Gemany Multicenter RCTs	CHD patients in cardiac rehabilitation.	Intervention n=27<br><br>control n=28	Male Intervention: n=81.5%<br><br>control n=71.9%	Intervention: 60.81 (11.06)<br><br>Contol: 55.62 (10.05)	BDI and HADS	Intervention delivered by psychotherapist, 4-6 session (50min/session), An individual face to face therapy	Intervention: CBT<br>Control: Usual care	Intervention, BDI score:Baseline: 19.04(6.39)<br>1 mo: 12.34(7.69)<br><br>U.C. BDI score: Baseline: 21.25(5.43)<br>1 mo:15.29(7.65)	72%

Notes:

Abbreviations: HADS, Hospital Anxiety and Depression Scale; BDI-II, Beck Depression Inventory-II; PHQ-9, Patient Health Questionnaire-9; CDS, Cardiac Depression Scale; CABG, Coronary Artery Bypass Graft; AVR, Aorta Valve Replacement; MVR, Mitral Valve Repair/Replacement;CBGI-CHD, Cognitive-Behavioral Group Intervention for Coronary Heart Disease; CTG, Cognitive Therapy in Groups; iCBT, internet-based cognitive behavioral therapy; A.P, Angina Plan; PST, Problem-Solving Treatment.

Table S2. Search Strategy

**PUBMED**		
#1	coronary heart disease OR cardiac OR heart OR myocardial	2,249,813
#2	psychosocial intervention OR psychotherapy OR CBT OR cognitive behavior therapy	306,202
#3	depressive OR depression	594,647
#4	experimental OR randomized trial	2,859,111
#5	(#1 AND #2 AND 3# AND 4#)<br>(((coronary heart disease OR cardiac OR heart OR myocardial) AND (psychosocial intervention OR psychotherapy OR CBT OR cognitive behavior therapy)) AND (depressive OR depression)) AND (experimental OR randomized trial)	748
**SCOPUS**		
1	coronary heart disease OR cardiac OR heart OR myocardial	1,429,260
2	TITLE-ABS-KEY (psychosocial AND intervention OR psychotherapy OR cbt OR cognitive AND behavior AND therapy)	8,361
3	TITLE-ABS-KEY (depressive OR depression)	878,441
4	TITLE-ABS-KEY (experimental OR randomized AND trial)	1,168,810
5	(TITLE-ABS-KEY (experimental OR randomized AND trial)) AND (TITLE-ABS-KEY (depressive OR depression)) AND (TITLE-ABS-KEY (psychosocial AND intervention OR psychotherapy OR cbt OR cognitive AND behavior AND therapy)) AND (coronary AND heart AND disease OR cardiac OR heart OR myocardial)	68
**CINAHL**		
S1	"cardiac disease*" OR "cardiac disorder*" OR "heart disease*" OR "heart disorder*" OR "heart failure*" OR cardiac* OR myocardia "chronic disease*" OR "chronic* illness*" OR “coronary disease*” OR “myocardial ischemia*” OR “coronary syndrome*”	376,982
S2	MH(("Heart disease”+) OR (myocardium+) OR (“chronic disease"+) OR ("heart failure"+))	11,569
S3	S1 OR S2	380,703
S4	psychosocial intervention OR psychotherapy OR CBT OR cognitive behavior therapy	59,569
S5	MH(("psychosocial intervention”+) OR (psychotherapy +) OR (“CBT "+) OR ("cognitive behavior therapy "+))	6,536
S6	S4 OR S5	59,754
S7	"depressive disorder" OR depression OR “depressive symptoms”	207,897
S8	MH("depression"+)	801
S9	S7 OR S8	207,983
	S3 AND S6 AND S9	463
S10	((MH "Clinical Trials+") or (PT Clinical trial) or (TX clinic* n1 trial*) or TX ((singl* n1 blind*) or (singl* n1 mask*)) or TX ((doubl* n1 blind*) or (doubl* n1 mask*)) or TX ((tripl* n1 blind*) or (tripl* n1 mask*)) or TX ((trebl* n1 blind*) or (trebl* n1 mask*)) or (TX randomi* control* trial*) or (MH "Random Assignment") or (TX random* allocat*) or (TX placebo*) or (MH "Placebos") or (MH "Quantitative Studies") or (TX allocat* random*))	1,806,910
S11	S3 AND S6 AND S9 AND S10	272

**ACADEMIC SEARCH COMPLETE**		
S1	"cardiac disease*" OR "cardiac disorder*" OR "heart disease*" OR "heart disorder*" OR "heart failure*" OR cardiac* OR myocardia "chronic disease*" OR "chronic* illness*" OR "coronary disease*" OR "myocardial ischemia*" OR "coronary syndrome*"	638,528
S2	MH(("Heart disease"+) OR (myocardium+) OR ("chronic disease"+) OR ("heart failure"+))	128,959
S3	S1 OR S2	644,774
S4	"psychosocial intervention" OR psychotherapy OR CBT OR "cognitive behavior therapy"	157,427
S5	MH(("psychosocial intervention"+) OR (psychotherapy +) OR ("CBT "+) OR ("cognitive behavior therapy "+))	72,105
S6	S4 OR S5	157,427
S7	"depressive disorder" OR depression OR "depressive symptoms"	344,215
S8	MH("depression"+)	185,875
S9	S7 OR S8	344,215
S10	((MH "Clinical Trials+") or (PT Clinical trial) or (TX clinic* n1 trial*) or TX ((singl* n1 blind*) or (singl* n1 mask*)) or TX ((doubl* n1 blind*) or (doubl* n1 mask*)) or TX ((tripl* n1 blind*) or (tripl* n1 mask*)) or TX ((trebl* n1 blind*) or (trebl* n1 mask*)) or (TX randomi* control* trial*) or (MH "Random Assignment") or (TX random* allocat*) or (TX placebo*) or (MH "Placebos") or (MH "Quantitative Studies") or (TX allocat* random*))	1,326,613
S11	S3 AND S6 AND S9 AND S10	338
**PSYCINFO**		
1	coronary heart disease.mp.	4,601
2	cardiac.mp.	19,733
3	heart.mp. or exp Heart/	70,843
4	myocardial.mp.	7,078
5	psychosocial intervention.mp.	2,574
6	psychotherapy.mp. or exp Psychotherapy/	250,344
7	CBT.mp.	16,831
8	cognitive behavior therapy.mp. or exp Cognitive Behavior Therapy/	28,186
9	depressive.mp.	156,818
10	depression.mp.	370,759
11	experimental.mp.	223,260
12	randomized trial.mp.	6,850
13	1 or 2 or 3 or 4	80,811
14	5 or 6 or 7 or 8	273,929
15	9 or 10	397,646
16	11 or 12	229,577
17	13 and 14 and 15 and 16	52
**Google Scholar**		
1	(coronary heart disease OR cardiac OR heart OR myocardial) AND (psychosocial intervention OR psychotherapy OR CBT OR cognitive behavior therapy) AND (depressive OR depression) AND (experimental OR randomized trial)	64.100
2	The first 100 articles were taken based on relevance	100

Figure S1. The forest plot of the sensitivity analysis of included studies when low risk of bias studies excluded

Study or Subgroup	Experimental Mean	Experimental SD	Experimental Total	Control Mean	Control SD	Control Total	Weight	Std. Mean Difference IV, Fixed, 95% CI
Barth 2005	12.34	7.69	27	15.29	7.65	28	5.2%	-0.38 [-0.91, 0.15]
Dao 2011	15.9	5.1	48	23.4	11.6	48	8.4%	-0.83 [-1.25, -0.41]
Davidson 2013	10.74	8.49	73	14.79	9.13	77	14.0%	-0.46 [-0.78, -0.13]
Doering 2013	10.1	9.8	32	16.6	8.9	23	4.8%	-0.68 [-1.23, -0.13]
Humphries 2021	6.2	3.8	89	7.8	3.5	112	18.5%	-0.44 [-0.72, -0.16]
Nejati 2022	18.88	5.26	17	20.17	5.87	17	3.2%	-0.23 [-0.90, 0.45]
Norlund 2018	6.6	3.3	117	8	3.8	122	22.4%	-0.39 [-0.65, -0.14]
Rafanelli 2020	29.39	6.55	50	32.2	7.26	50	9.4%	-0.40 [-0.80, -0.01]
Turner 2013	20.5	8.4	21	19.6	9.3	31	4.8%	0.10 [-0.46, 0.65]
Zetta 2011	6.72	4.62	44	5.82	4.15	54	9.2%	0.20 [-0.19, 0.60]
**Total (95% CI)**			**518**			**562**	**100.0%**	**-0.38 [-0.50, -0.26]**

Heterogeneity: Chi² = 17.30, df = 9 (P = 0.04); I² = 48%
Test for overall effect: Z = 6.09 (P < 0.00001)

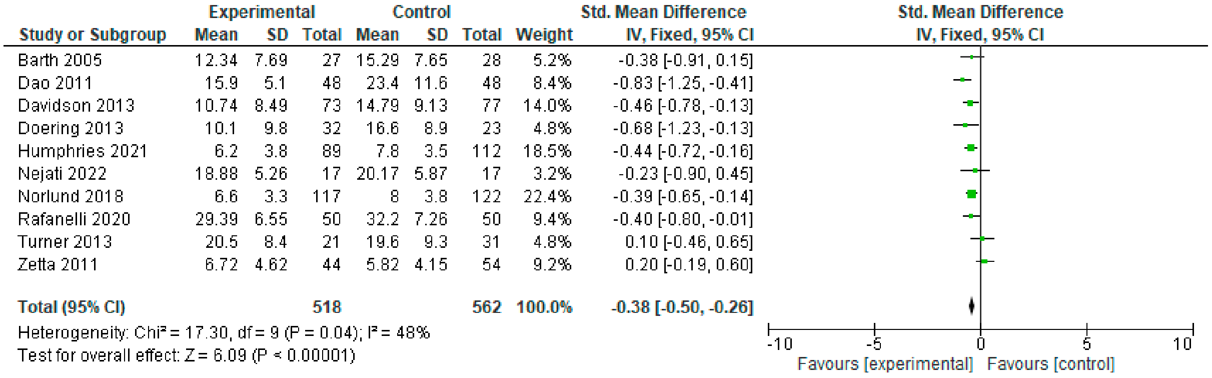

Figure S2. The forest plot of the sensitivity analysis of included studies when high risk of bias studies excluded

Study or Subgroup	Experimental Mean	Experimental SD	Experimental Total	Control Mean	Control SD	Control Total	Weight	Std. Mean Difference IV, Fixed, 95% CI
Berkman 2003	9.1	8.6	1238	12.2	9.1	1234	70.9%	-0.35 [-0.43, -0.27]
Davidson 2013	10.74	8.49	73	14.79	9.13	77	4.3%	-0.46 [-0.78, -0.13]
Freedland 2009	5.4	8.32	41	13.8	8.85	40	2.1%	-0.97 [-1.43, -0.51]
Humphries 2021	6.2	3.8	89	7.8	3.5	112	5.6%	-0.44 [-0.72, -0.16]
Norlund 2018	6.6	3.3	117	8	3.8	122	6.8%	-0.39 [-0.65, -0.14]
O'Neil 2014	6.1	5.5	53	8.11	5.8	53	3.0%	-0.35 [-0.74, 0.03]
O'Neil 2015	6.5	4.9	28	9.3	7.7	28	1.6%	-0.43 [-0.96, 0.10]
Rafanelli 2020	29.39	6.55	50	32.2	7.26	50	2.9%	-0.40 [-0.80, -0.01]
Zetta 2011	6.72	4.62	44	5.82	4.15	54	2.8%	0.20 [-0.19, 0.60]
**Total (95% CI)**			**1733**			**1770**	**100.0%**	**-0.36 [-0.43, -0.30]**

Heterogeneity: Chi² = 15.23, df = 8 (P = 0.05); I² = 47%
Test for overall effect: Z = 10.63 (P < 0.00001)

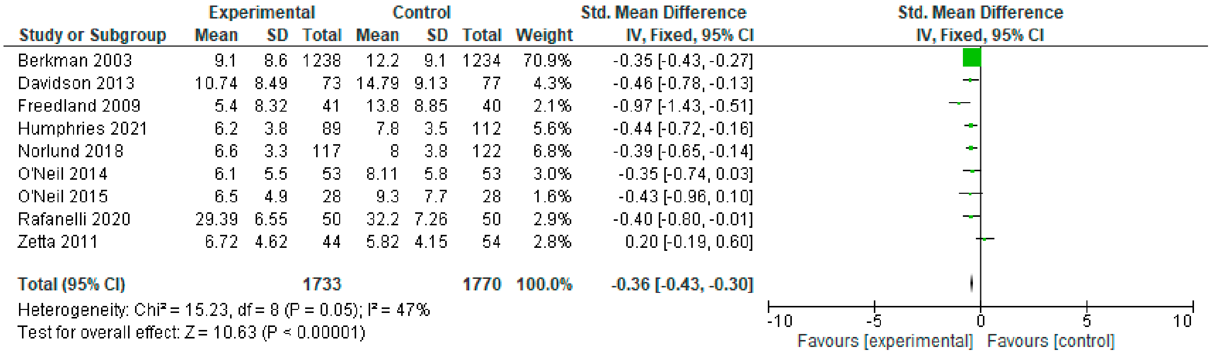

Figure S3. The forest plot of included studies with outlier

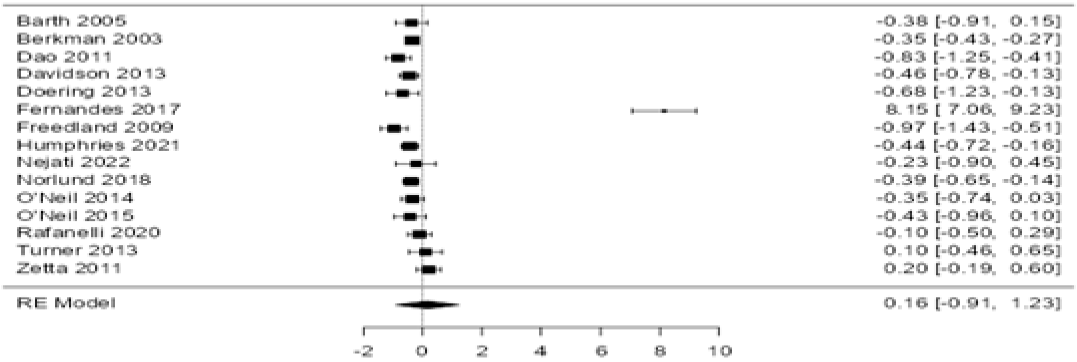

Figure S4. The funnel plot of included studies with outlier

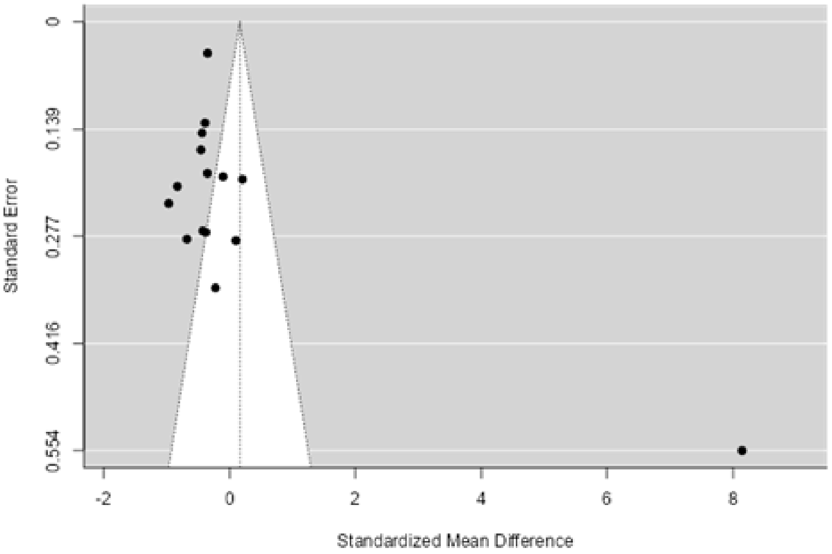

Figure S5. Effect of CBT Based on Short-Term Follow-Up (≤ 3 months).

	Experimental			Control				Std. Mean Difference
**Study or Subgroup**	**Mean**	**SD**	**Total**	**Mean**	**SD**	**Total**	**Weight**	**IV, Random, 95% CI**
Barth 2005	12.34	7.69	27	15.29	7.65	28	10.8%	-0.38 [-0.91, 0.15]
Dao 2011	15.9	5.1	48	23.4	11.6	48	13.9%	-0.83 [-1.25, -0.41]
Doering 2013	10.1	9.8	32	16.6	8.9	23	10.4%	-0.68 [-1.23, -0.13]
Freedland 2009	5.4	8.32	41	13.8	8.85	40	12.6%	-0.97 [-1.43, -0.51]
Nejati 2022	18.88	5.26	17	20.17	5.87	17	8.0%	-0.23 [-0.90, 0.45]
Norlund 2018	6.6	3.3	117	8	3.8	122	19.4%	-0.39 [-0.65, -0.14]
Rafanelli 2020	30.48	5.81	50	31.89	7.11	50	14.6%	-0.22 [-0.61, 0.18]
Turner 2013	20.5	8.4	21	19.6	9.3	31	10.3%	0.10 [-0.46, 0.65]
**Total (95% CI)**			**353**			**359**	**100.0%**	**-0.46 [-0.69, -0.23]**

Heterogeneity: Tau² = 0.05; Chi² = 14.51, df = 7 (P = 0.04); I² = 52%
Test for overall effect: Z = 3.95 (P < 0.0001)

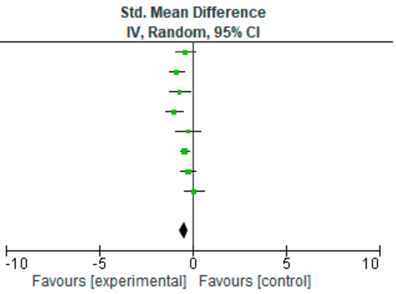

Figure S6. Effect of CBT based on Middle-Term Follow-Up (post-6 months intervention).

	Experiment			Control				Std. Mean Difference
**Study or Subgroup**	**Mean**	**SD**	**Total**	**Mean**	**SD**	**Total**	**Weight**	**IV, Random, 95% CI**
Berkman 2003	9.1	8.6	1238	12.2	9.1	1234	17.1%	-0.35 [-0.43, -0.27]
Davidson 2013	10.74	8.49	73	14.79	9.13	77	15.1%	-0.46 [-0.78, -0.13]
Freedland 2009	7.8	1.3	41	10.7	1.4	40	12.3%	-2.13 [-2.68, -1.58]
O'Neil 2014	6.1	5.5	53	8.11	5.8	53	14.4%	-0.35 [-0.74, 0.03]
Rafanelli 2020	29.89	5.88	50	30.59	7.28	50	14.3%	-0.10 [-0.50, 0.29]
Turner 2013	19.8	10.3	20	17.8	9.5	28	12.0%	0.20 [-0.38, 0.78]
Zetta 2011	6.72	4.62	44	5.82	4.15	102	14.8%	0.21 [-0.15, 0.56]
**Total (95% CI)**			**1519**			**1584**	**100.0%**	**-0.40 [-0.76, -0.04]**

Heterogeneity: Tau² = 0.19; Chi² = 55.06, df = 6 (P < 0.00001); I² = 89%
Test for overall effect: Z = 2.19 (P = 0.03)

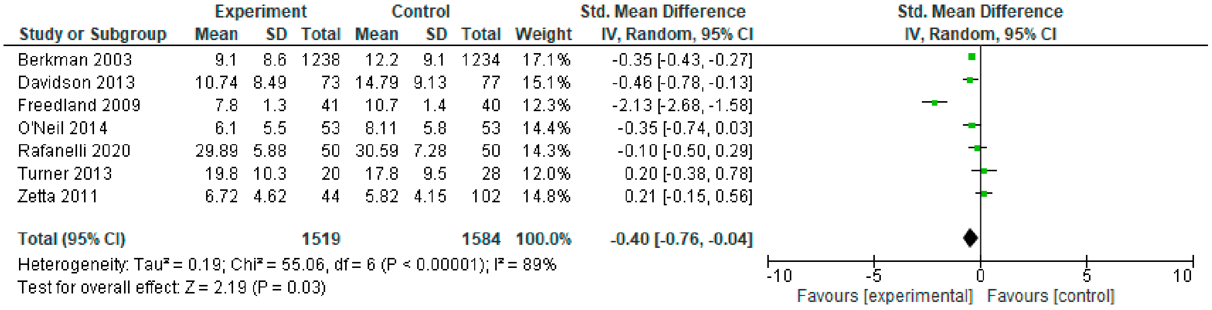

Figure S7. Effect of CBT Based on Long-Term Follow-Up (≥ 12 months).

Study or Subgroup	Experiment Mean	Experiment SD	Experiment Total	Control Mean	Control SD	Control Total	Weight	Std. Mean Difference IV, Random, 95% CI
Freedland 2009	6.7	1.3	41	12.9	1.4	40	18.6%	-4.55 [-5.39, -3.71]
Humphries 2021	6.2	3.8	89	7.8	3.5	112	20.9%	-0.44 [-0.72, -0.16]
O'Neil 2015	6.5	4.9	28	9.3	7.7	28	20.1%	-0.43 [-0.96, 0.10]
Rafanelli 2020	29.7	6.51	50	30.03	7.05	50	20.6%	-0.05 [-0.44, 0.34]
Turner 2013	17.4	10.3	20	16.6	10.5	27	19.9%	0.08 [-0.50, 0.65]
**Total (95% CI)**			**228**			**257**	**100.0%**	**-1.02 [-2.05, 0.01]**

Heterogeneity: Tau^2 = 1.31; Chi^2 = 98.41, df = 4 (P < 0.00001); I^2 = 96%
Test for overall effect: Z = 1.93 (P = 0.05)

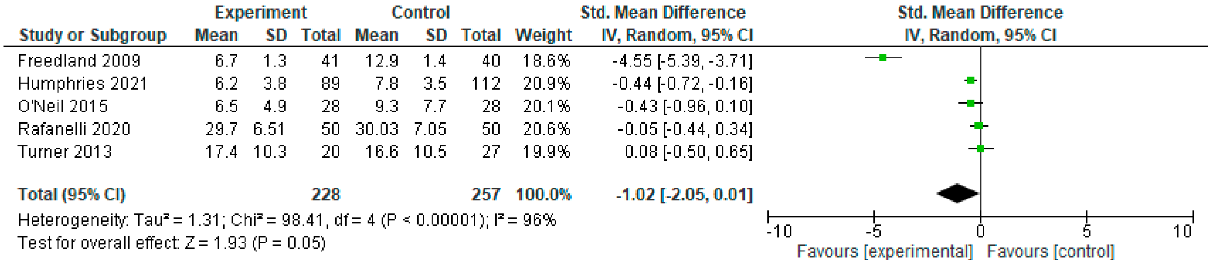

Figure S8. Effect of CBT Based on Face-to-Face Delivery Mode.

Study or Subgroup	Experimental Mean	Experimental SD	Experimental Total	Control Mean	Control SD	Control Total	Weight	Std. Mean Difference IV, Fixed, 95% CI
Barth 2005	12.34	7.69	27	15.29	7.65	28	1.8%	-0.38 [-0.91, 0.15]
Berkman 2003	9.1	8.6	1238	12.2	9.1	1234	82.3%	-0.35 [-0.43, -0.27]
Dao 2011	15.9	5.1	48	23.4	11.6	48	3.0%	-0.83 [-1.25, -0.41]
Davidson 2013	10.74	8.49	73	14.79	9.13	77	4.9%	-0.46 [-0.78, -0.13]
Doering 2013	10.1	9.8	32	16.6	8.9	23	1.7%	-0.68 [-1.23, -0.13]
Nejati 2022	18.88	5.26	17	20.17	5.87	17	1.1%	-0.23 [-0.90, 0.45]
Rafanelli 2020	29.89	5.88	50	30.59	7.28	50	3.4%	-0.10 [-0.50, 0.29]
Turner 2013	20.5	8.4	21	19.6	9.3	31	1.7%	0.10 [-0.46, 0.65]
**Total (95% CI)**			**1506**			**1508**	**100.0%**	**-0.36 [-0.43, -0.29]**

Heterogeneity: Chi^2 = 10.97, df = 7 (P = 0.14); I^2 = 36%
Test for overall effect: Z = 9.75 (P < 0.00001)

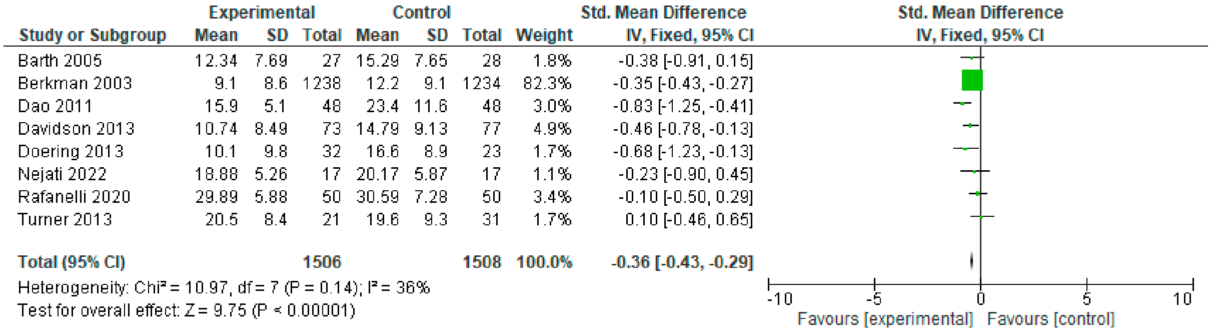

Figure S9. Effect of CBT Based on Remote (By Phone or Internet) Delivery Mode.

Study or Subgroup	Experimental Mean	Experimental SD	Experimental Total	Control Mean	Control SD	Control Total	Weight	Std. Mean Difference IV, Fixed, 95% CI
Humphries 2021	6.2	3.8	89	7.8	3.5	112	28.3%	-0.44 [-0.72, -0.16]
Norlund 2018	6.6	3.3	117	8	3.8	122	34.3%	-0.39 [-0.65, -0.14]
O'Neil 2014	6.1	5.5	53	8.11	5.8	53	15.3%	-0.35 [-0.74, 0.03]
O'Neil 2015	6.5	4.9	28	9.3	7.7	28	8.0%	-0.43 [-0.96, 0.10]
Zetta 2011	6.72	4.62	44	5.82	4.15	54	14.1%	0.20 [-0.19, 0.60]
**Total (95% CI)**			**331**			**369**	**100.0%**	**-0.32 [-0.47, -0.17]**

Heterogeneity: Chi^2 = 7.80, df = 4 (P = 0.10); I^2 = 49%
Test for overall effect: Z = 4.15 (P < 0.0001)

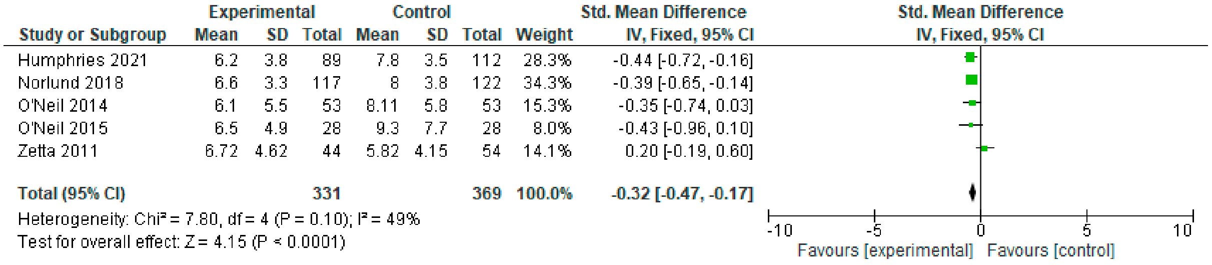

Figure S10. Effect of CBT Based on Internet Delivery Mode.

Study or Subgroup	Experimental Mean	Experimental SD	Experimental Total	Control Mean	Control SD	Control Total	Weight	Std. Mean Difference IV, Fixed, 95% CI
Humphries 2021	6.2	3.8	89	7.8	3.5	112	45.3%	-0.44 [-0.72, -0.16]
Norlund 2018	6.6	3.3	117	8	3.8	122	54.7%	-0.39 [-0.65, -0.14]
**Total (95% CI)**			**206**			**234**	**100.0%**	**-0.41 [-0.60, -0.22]**

Heterogeneity: Chi^2 = 0.06, df = 1 (P = 0.81); I^2 = 0%
Test for overall effect: Z = 4.27 (P < 0.0001)

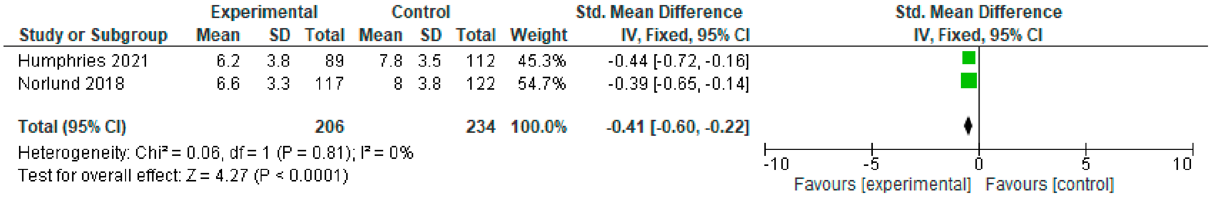

Figure S11. Effect of CBT Based on The Low Frequency of Meetings (3 – 6 Session).

Study or Subgroup	Experimental Mean	Experimental SD	Experimental Total	Control Mean	Control SD	Control Total	Weight	Std. Mean Difference IV, Random, 95% CI
Barth 2005	12.34	7.69	27	15.29	7.65	28	32.1%	-0.38 [-0.91, 0.15]
Dao 2011	15.9	5.1	48	23.4	11.6	48	36.6%	-0.83 [-1.25, -0.41]
Turner 2013	20.5	8.4	21	19.6	9.3	31	31.3%	0.10 [-0.46, 0.65]
**Total (95% CI)**			**96**			**107**	**100.0%**	**-0.39 [-0.93, 0.15]**

Heterogeneity: Tau^2 = 0.16; Chi^2 = 7.02, df = 2 (P = 0.03); I^2 = 71%
Test for overall effect: Z = 1.43 (P = 0.15)

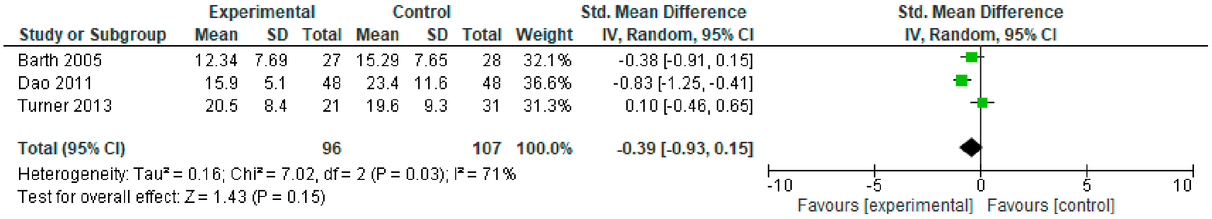

Figure S12. Effect of CBT Based on The Medium Frequency of Meetings (8 – 12 Sessions).

Study or Subgroup	Experimental Mean	Experimental SD	Experimental Total	Control Mean	Control SD	Control Total	Weight	Std. Mean Difference IV, Random, 95% CI
Doering 2013	10.1	9.8	32	16.6	8.9	23	12.8%	-0.68 [-1.23, -0.13]
Freedland 2009	5.4	8.32	41	13.8	8.85	40	14.7%	-0.97 [-1.43, -0.51]
Nejati 2022	18.88	5.26	17	20.17	5.87	17	10.5%	-0.23 [-0.90, 0.45]
O'Neil 2014	6.1	5.5	53	8.11	5.8	53	16.4%	-0.35 [-0.74, 0.03]
O'Neil 2015	6.5	4.9	28	9.3	7.7	28	13.2%	-0.43 [-0.96, 0.10]
Rafanelli 2020	29.89	5.88	50	30.59	7.28	50	16.2%	-0.10 [-0.50, 0.29]
Zetta 2011	6.72	4.62	44	5.82	4.15	54	16.1%	0.20 [-0.19, 0.60]
**Total (95% CI)**			**265**			**265**	**100.0%**	**-0.35 [-0.65, -0.05]**

Heterogeneity: Tau² = 0.10; Chi² = 17.27, df = 6 (P = 0.008); I² = 65%
Test for overall effect: Z = 2.29 (P = 0.02)

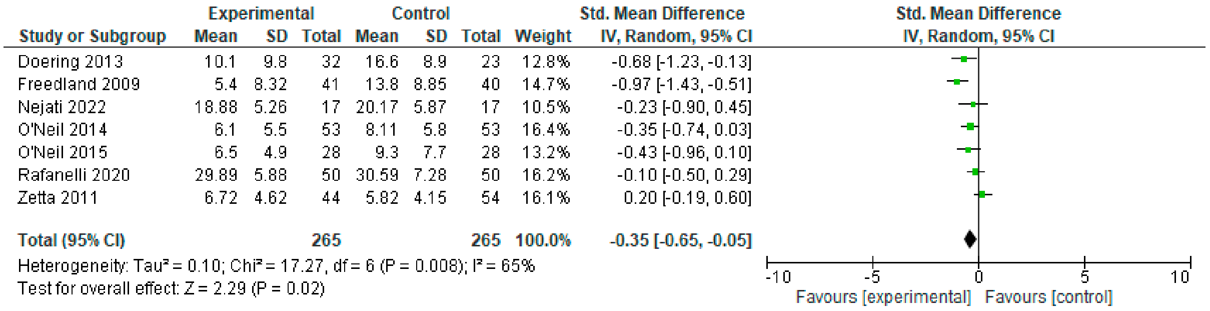

Figure S13. Effect of CBT Based on Frequent Meetings (≥ 13 Sessions).

Study or Subgroup	Experimental Mean	Experimental SD	Experimental Total	Control Mean	Control SD	Control Total	Weight	Std. Mean Difference IV, Fixed, 95% CI
Berkman 2003	9.1	8.6	1238	12.2	9.1	1234	85.0%	-0.35 [-0.43, -0.27]
Humphries 2021	6.2	3.8	89	7.8	3.5	112	6.8%	-0.44 [-0.72, -0.16]
Norlund 2018	6.6	3.3	117	8	3.8	122	8.2%	-0.39 [-0.65, -0.14]
**Total (95% CI)**			**1444**			**1468**	**100.0%**	**-0.36 [-0.43, -0.29]**

Heterogeneity: Chi² = 0.42, df = 2 (P = 0.81); I² = 0%
Test for overall effect: Z = 9.62 (P < 0.00001)

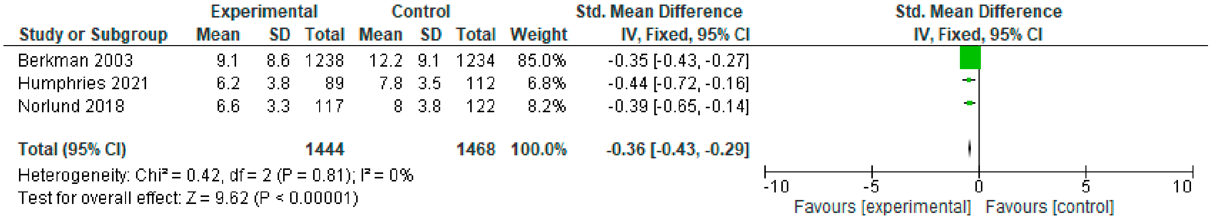

Figure S14. Effect of CBT Based on Individual Delivery Mode

Study or Subgroup	Experimental Mean	Experimental SD	Experimental Total	Control Mean	Control SD	Control Total	Weight	Std. Mean Difference IV, Random, 95% CI
Zetta 2011	6.72	4.62	44	5.82	4.15	54	11.5%	0.20 [-0.19, 0.60]
O'Neil 2015	6.5	4.9	28	9.3	7.7	28	11.0%	-0.43 [-0.96, 0.10]
O'Neil 2014	6.1	5.5	53	8.11	5.8	53	11.6%	-0.35 [-0.74, 0.03]
Norlund 2018	6.6	3.3	117	8	3.8	122	12.0%	-0.39 [-0.65, -0.14]
Humphries 2021	6.2	3.8	89	7.8	3.5	112	11.9%	-0.44 [-0.72, -0.16]
Freedland 2009	5.4	1.3	41	13.8	1.4	40	8.3%	-6.16 [-7.23, -5.10]
Doering 2013	10.1	9.8	32	16.6	8.9	36	11.2%	-0.69 [-1.18, -0.20]
Dao 2011	15.9	5.1	48	23.4	11.6	48	11.5%	-0.83 [-1.25, -0.41]
Barth 2005	12.34	7.69	27	15.29	7.65	28	11.0%	-0.38 [-0.91, 0.15]
**Total (95% CI)**			**479**			**521**	**100.0%**	**-0.89 [-1.43, -0.35]**

Heterogeneity: Tau² = 0.62; Chi² = 124.98, df = 8 (P < 0.00001); I² = 94%
Test for overall effect: Z = 3.23 (P = 0.001)

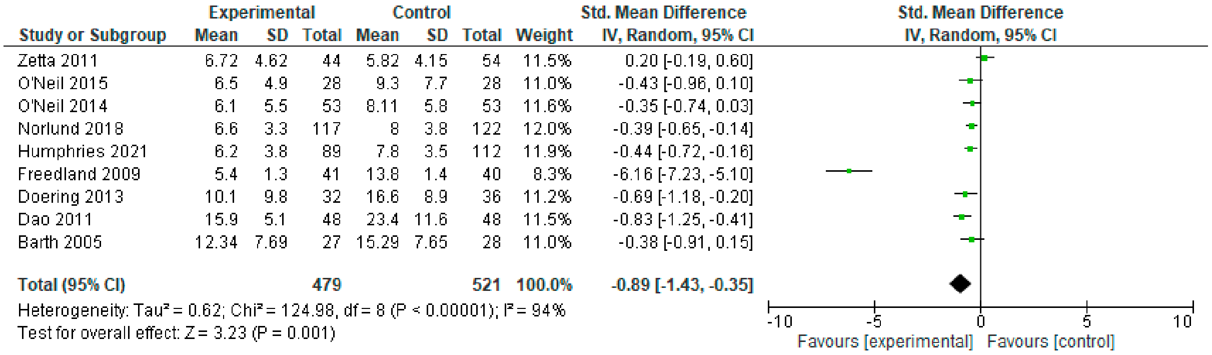

Figure S15. Effect of CBT Based on Mixed (Group and Individual) Delivery Mode.

Study or Subgroup	Experimental Mean	Experimental SD	Experimental Total	Control Mean	Control SD	Control Total	Weight	Std. Mean Difference IV, Fixed, 95% CI
Berkman 2003	9.1	8.6	1238	12.2	9.1	1234	94.3%	-0.35 [-0.43, -0.27]
Davidson 2013	10.74	8.49	73	14.79	9.13	77	5.7%	-0.46 [-0.78, -0.13]
**Total (95% CI)**			**1311**			**1311**	**100.0%**	**-0.36 [-0.43, -0.28]**

Heterogeneity: Chi² = 0.39, df = 1 (P = 0.53); I² = 0%
Test for overall effect: Z = 9.04 (P < 0.00001)

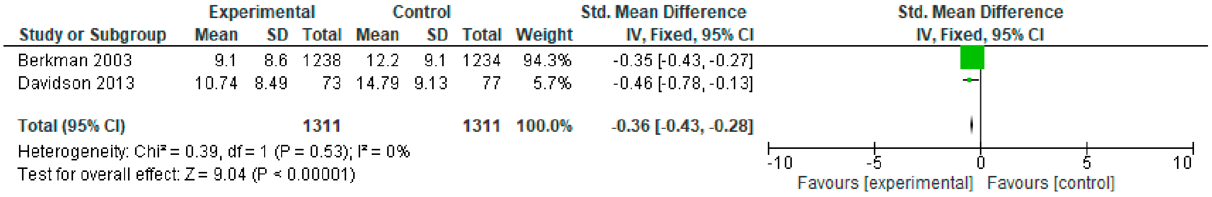

Figure S16. Effect of CBT based on Individual Delivery Mode After Two Studies with CABG Patients was Issued.

Study or Subgroup	Experimental Mean	SD	Total	Control Mean	SD	Total	Weight	Std. Mean Difference IV, Fixed, 95% CI
Barth 2005	12.34	7.69	27	15.29	7.65	28	6.8%	-0.38 [-0.91, 0.15]
Doering 2013	10.1	9.8	32	16.6	8.9	23	6.4%	-0.68 [-1.23, -0.13]
Humphries 2021	6.2	3.8	89	7.8	3.5	112	24.6%	-0.44 [-0.72, -0.16]
Norlund 2018	6.6	3.3	117	8	3.8	122	29.7%	-0.39 [-0.65, -0.14]
O'Neil 2014	6.1	5.5	53	8.11	5.8	53	13.2%	-0.35 [-0.74, 0.03]
O'Neil 2015	6.5	4.9	28	9.3	7.7	28	6.9%	-0.43 [-0.96, 0.10]
Zetta 2011	6.72	4.62	44	5.82	4.15	54	12.2%	0.20 [-0.19, 0.60]
**Total (95% CI)**			**390**			**420**	**100.0%**	**-0.35 [-0.48, -0.21]**

Heterogeneity: Chi² = 9.35, df = 6 (P = 0.15); I² = 36%
Test for overall effect: Z = 4.84 (P < 0.00001)

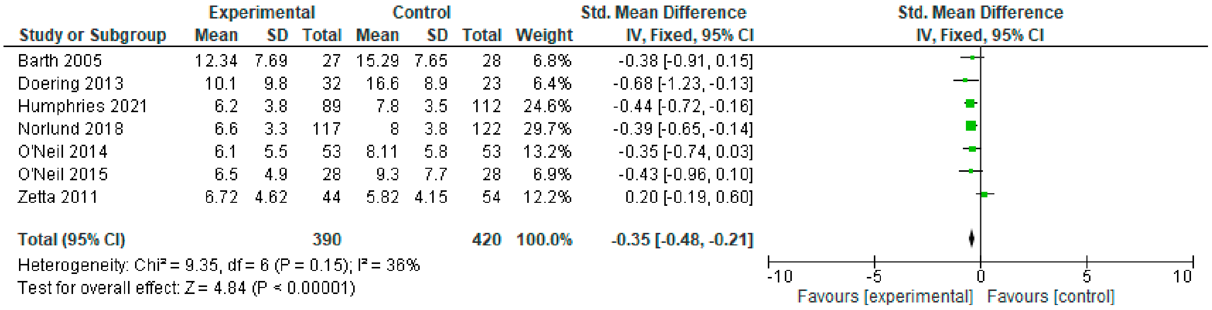

Figure S17. Effect of CBT Based on Group Delivery Mode**.**

Study or Subgroup	Experimental Mean	SD	Total	Control Mean	SD	Total	Weight	Std. Mean Difference IV, Fixed, 95% CI
Nejati 2022	18.88	5.26	17	20.17	5.87	17	18.4%	-0.23 [-0.90, 0.45]
Rafanelli 2020	29.89	5.88	50	30.59	7.28	50	54.4%	-0.10 [-0.50, 0.29]
Turner 2013	20.5	8.4	21	19.6	9.3	31	27.2%	0.10 [-0.46, 0.65]
**Total (95% CI)**			**88**			**98**	**100.0%**	**-0.07 [-0.36, 0.22]**

Heterogeneity: Chi² = 0.59, df = 2 (P = 0.74); I² = 0%
Test for overall effect: Z = 0.49 (P = 0.63)

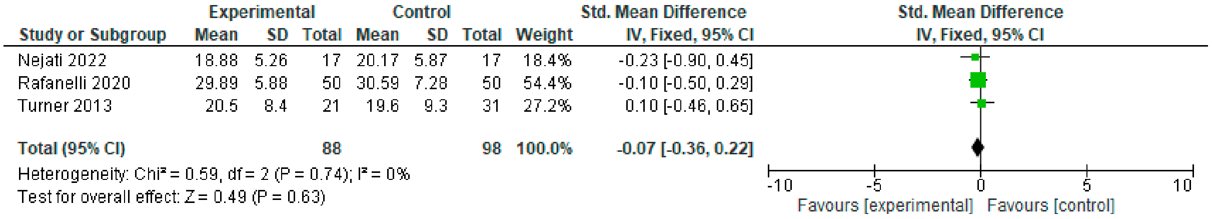

Figure S18. Effect of CBT without Combination.

Study or Subgroup	Experimental Mean	SD	Total	Control Mean	SD	Total	Weight	Std. Mean Difference IV, Random, 95% CI
Barth 2005	12.34	7.69	27	15.29	7.65	28	8.9%	-0.38 [-0.91, 0.15]
Dao 2011	15.9	5.1	48	23.4	11.6	48	10.9%	-0.83 [-1.25, -0.41]
Doering 2013	10.1	9.8	32	16.6	8.9	23	8.6%	-0.68 [-1.23, -0.13]
Freedland 2009	5.4	8.32	41	13.8	8.85	40	10.1%	-0.97 [-1.43, -0.51]
Nejati 2022	18.88	5.26	17	20.17	5.87	17	6.9%	-0.23 [-0.90, 0.45]
Norlund 2018	6.6	3.3	117	8	3.8	122	14.2%	-0.39 [-0.65, -0.14]
O'Neil 2014	6.1	5.5	53	8.11	5.8	53	11.6%	-0.35 [-0.74, 0.03]
O'Neil 2015	6.5	4.9	28	9.3	7.7	28	8.9%	-0.43 [-0.96, 0.10]
Turner 2013	20.5	8.4	21	19.6	9.3	31	8.6%	0.10 [-0.46, 0.65]
Zetta 2011	6.72	4.62	44	5.82	4.15	54	11.3%	0.20 [-0.19, 0.60]
**Total (95% CI)**			**428**			**444**	**100.0%**	**-0.40 [-0.63, -0.17]**

Heterogeneity: Tau² = 0.08; Chi² = 23.15, df = 9 (P = 0.006); I² = 61%
Test for overall effect: Z = 3.43 (P = 0.0006)

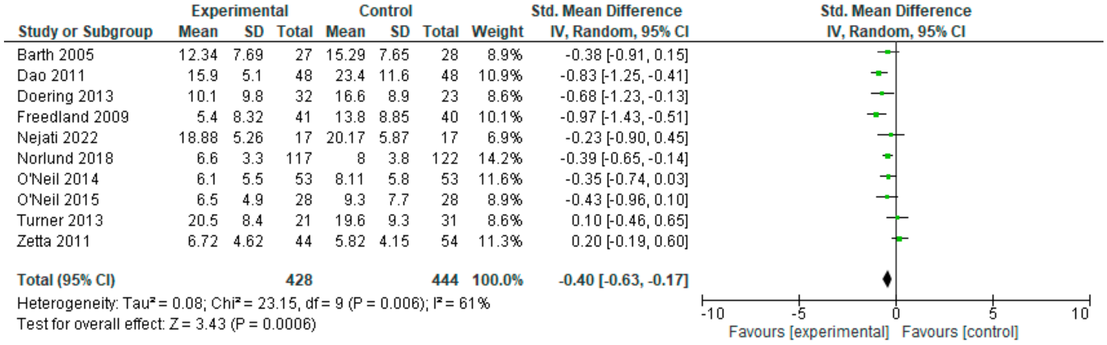

Figure S19. Effect of CBT Combined with other Therapy.

Study or Subgroup	Experimental Mean	SD	Total	Control Mean	SD	Total	Weight	Std. Mean Difference IV, Fixed, 95% CI
Berkman 2003	9.1	8.6	1238	12.2	9.1	1234	84.8%	-0.35 [-0.43, -0.27]
Davidson 2013	10.74	8.49	73	14.79	9.13	77	5.1%	-0.46 [-0.78, -0.13]
Humphries 2021	6.2	3.8	89	7.8	3.5	112	6.7%	-0.44 [-0.72, -0.16]
Rafanelli 2020	29.39	6.55	50	32.2	7.26	50	3.4%	-0.40 [-0.80, -0.01]
**Total (95% CI)**			**1450**			**1473**	**100.0%**	**-0.36 [-0.44, -0.29]**

Heterogeneity: Chi² = 0.74, df = 3 (P = 0.86); I² = 0%
Test for overall effect: Z = 9.73 (P < 0.00001)

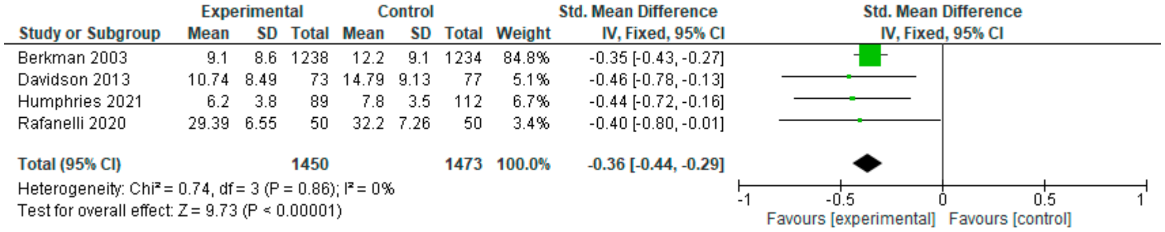

Figure S20. Effect of CBT on Patients with CABG.

Study or Subgroup	Experimental Mean	SD	Total	Control Mean	SD	Total	Weight	Std. Mean Difference IV, Fixed, 95% CI
Dao 2011	15.9	5.1	48	23.4	11.6	48	41.8%	-0.83 [-1.25, -0.41]
Doering 2013	10.1	9.8	32	16.6	8.9	23	24.0%	-0.68 [-1.23, -0.13]
Freedland 2009	5.4	8.32	41	13.8	8.85	40	34.2%	-0.97 [-1.43, -0.51]
**Total (95% CI)**			**121**			**111**	**100.0%**	**-0.84 [-1.11, -0.57]**

Heterogeneity: Chi² = 0.63, df = 2 (P = 0.73); I² = 0%
Test for overall effect: Z = 6.11 (P < 0.00001)

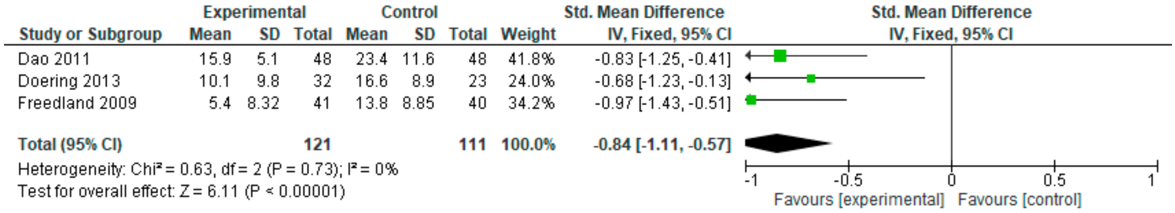

Figure S21. Effect of CBT on Patients with Various Treatments

	Experimental			Control				Std. Mean Difference
Study or Subgroup	Mean	SD	Total	Mean	SD	Total	Weight	IV, Fixed, 95% CI
Barth 2005	12.34	7.69	27	15.29	7.65	28	1.5%	-0.38 [-0.91, 0.15]
Berkman 2003	9.1	8.6	1238	12.2	9.1	1234	69.6%	-0.35 [-0.43, -0.27]
Davidson 2013	10.74	8.49	73	14.79	9.13	77	4.2%	-0.46 [-0.78, -0.13]
Humphries 2021	6.2	3.8	89	7.8	3.5	112	5.5%	-0.44 [-0.72, -0.16]
Nejati 2022	18.88	5.26	17	20.17	5.87	17	1.0%	-0.23 [-0.90, 0.45]
Norlund 2018	6.6	3.3	117	8	3.8	122	6.7%	-0.39 [-0.65, -0.14]
O'Neil 2014	6.1	5.5	53	8.11	5.8	53	3.0%	-0.35 [-0.74, 0.03]
O'Neil 2015	6.5	4.9	28	9.3	7.7	28	1.6%	-0.43 [-0.96, 0.10]
Rafanelli 2020	29.39	6.55	50	32.2	7.26	50	2.8%	-0.40 [-0.80, -0.01]
Turner 2013	20.5	8.4	21	19.6	9.3	31	1.4%	0.10 [-0.46, 0.65]
Zetta 2011	6.72	4.62	44	5.82	4.15	54	2.8%	0.20 [-0.19, 0.60]
**Total (95% CI)**			**1757**			**1806**	**100.0%**	**-0.34 [-0.41, -0.28]**

Heterogeneity: $Chi^2 = 11.07$, $df = 10$ ($P = 0.35$); $I^2 = 10\%$
Test for overall effect: $Z = 10.13$ ($P < 0.00001$)

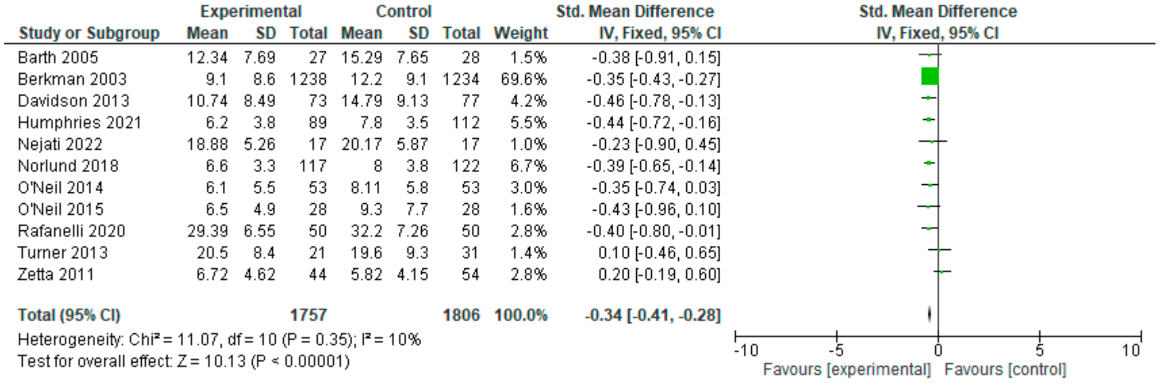

Supplement: Supplementary file 1 [file healthcare-11-00943-s001.zip › healthcare-2202733-supplementary.pdf]
